# Affective Reactivity to Daily Romantic Relationship Tension at Midlife and Older Adulthood

**DOI:** 10.1093/geroni/igaf122.3062

**Published:** 2025-12-31

**Authors:** Jeesun Lee, David Almeida, Steffany Fredman

**Affiliations:** The Pennsylvania State University, University Park, Pennsylvania, United States; The Pennsylvania State University, University Park, Pennsylvania, United States; The Pennsylvania State University, University Park, Pennsylvania, United States

## Abstract

Affective reactivity to daily stressors is a marker of psychological vulnerability at midlife and older adulthood. However, little is known about affective reactivity to daily relationship tension specifically and whether this reactivity depends on global romantic relationship quality and gender. Using data from the National Study of Daily Experiences (N = 2,804; Mage = 53.31, range = 33 – 83; 51.7% female), we investigated the daily linkages between relationship tension (e.g., argument or disagreement) and same day negative affect (NA) and positive affect (PA) in an 8-day daily diary study, examining whether global relationship strain, support, and gender moderated these associations. Multilevel modeling revealed main effects at the between- and within-person levels. Individuals who experienced greater relationship tension reported higher NA and lower PA, on average, and on days when tension was higher, they reported elevated NA and lower PA. Moderation analyses indicated that those with more strained or less supportive relationships showed stronger NA reactivity to daily relationship tension. In addition, although men and women did not differ in affective reactivity to relationship tension, women with more strained relationships had higher average NA than men, suggesting that the cumulative effects of relationship tension may be worse for women’s emotional well-being. These findings highlight the link between daily relationship tension and NA at midlife, particularly among individuals with lower quality romantic relationships. Interventions that reduce global relationship strain and promote relationship support may protect health and well-being in midlife and older adulthood by reducing affectivity reactivity to daily relationship tension.

